# Milk Fatty Acid Profile in Response to Acute Underfeeding in Dairy Sheep Divergent for Feed Efficiency

**DOI:** 10.3390/ani16030426

**Published:** 2026-01-29

**Authors:** Esther Barrio, Clàudia Baila, Pablo A. S. Fonseca, Pablo G. Toral, Pilar Frutos, Gonzalo Hervás

**Affiliations:** Instituto de Ganadería de Montaña, CSIC-University of León, Finca Marzanas s/n, Grulleros, 24346 León, Spain; e.barrio@csic.es (E.B.); c.baila@csic.es (C.B.); p.fonseca@csic.es (P.A.S.F.); pablo.toral@csic.es (P.G.T.); p.frutos@csic.es (P.F.)

**Keywords:** milk lipids, nutritional challenge, residual feed intake, sheep

## Abstract

Milk fatty acid (FA) composition derives from de novo synthesis in the mammary gland and from the uptake of preformed FA derived from diet, ruminal biohydrogenation, and body tissue mobilization. Consequently, milk FA profiles have been related to nutritional stress responses and feed efficiency (FE). This study tested whether dairy sheep with divergent FE would show different milk FA responses to a nutritional challenge. After calculating two FE indices in 40 ewes, the 9 most efficient (H-FE) and 9 least efficient (L-FE) animals were selected and subjected to a 3-day challenge replacing their total mixed ration (TMR) with wheat straw, followed by TMR refeeding. Temporal patterns of milk FA variation showed only minor differences between H-FE and L-FE ewes. The L-FE group exhibited higher concentrations of *iso*- and *anteiso*-17:0 and 18:0, suggesting differences in rumen fermentation and biohydrogenation. In contrast, underfeeding induced marked changes in milk FA composition, including a strong reduction in short- and medium-chain FAs (≤C16), consistent with a shift from de novo synthesis toward increased uptake of preformed FA. Overall, limited differences suggest that milk FA composition may not be a suitable marker for FE. Nonetheless, underfeeding-induced changes highlight its value for exploring nutritional stress responses.

## Simple Summary

This study tested the hypothesis that dairy Assaf sheep with divergent feed efficiency (FE) would respond differently to a nutritional challenge, and this different response would be reflected in their milk fatty acid (FA) composition. Therefore, changes in FA profile may serve as an indicator of FE. To explore this, nine high- and nine low-FE lactating ewes were subjected to a nutritional challenge consisting of feeding them with only wheat straw for 3 days. This feed restriction led to a strong response in milk FA profile (especially, a marked reduction in short- and medium-chain FAs of de novo synthesis). However, limited differences were observed between FE groups, which suggests that, in the current scenario, milk FA composition might not be a suitable marker for clustering more and less feed-efficient dairy ewes. Nonetheless, changes in the milk FA profile due to underfeeding highlight its value for exploring the response to nutritional stress.

## 1. Introduction

Milk fat contains numerous fatty acids (FA) originating from either de novo synthesis in the mammary tissue or uptake of preformed FA from plasma. De novo synthesis generates short- and medium-chain milk FA (i.e., most FAs with 16 or fewer carbons) using volatile fatty acids (VFAs) produced during ruminal fermentation [1,2]. In contrast, preformed FAs (i.e., most FAs with 16 or more carbons) are derived from multiple sources, such as dietary FA, ruminal biohydrogenation, and body tissue mobilization (mainly adipose tissue) [1,2].

The relative contributions of de novo and preformed FA to milk fat may vary depending on animals’ feed efficiency (FE) [3,4]. Differences in FE have been associated with variations in rumen fermentation [5,6], which may affect the outflow dynamics of fermentation products, modifying the availability of substrates for mammary lipogenesis and thus milk FA composition [7,8,9]. Differences in FE have also been related to different responses to nutritional conditions (energy balance) and fat mobilization [1,9]. For example, a negative energy balance increases body fat mobilization, altering the proportions of de novo vs. preformed FA in milk fat [10]. On this basis, given the limited knowledge about the ability of animals with divergent FE to cope with nutritional challenges, it is relevant to investigate their responses. Such challenges are expected to become more frequent under a possible scenario involving climate change, high raw material prices, and increasing feed–food competition. Therefore, subjecting more and less efficient sheep to an acute feed restriction may reveal changes in milk FA composition that serve as a dynamic indicator of how they manage energy resources under stress [11,12]. This is important because enhancing FE is a key goal in dairy production to lower feed costs, increase farm profit, and reduce the environmental footprint [13,14]. However, FE is a highly complex trait, with considerable individual variability among animals [15,16], and its accurate estimation is difficult as direct measurements are labor- or resource-demanding. In this context, identifying reliable indirect indicators, such as milk FA, would be of utmost interest [17,18].

By integrating both individual differences in FE and responses to underfeeding, we could gain deeper insights into metabolic and physiological mechanisms underlying FE. The response and recovery of low- and high-FE dairy ewes to an acute feed restriction in terms of productive performance, blood metabolites, and ruminal fermentation has already been assessed by our team, as reported by Barrio et al. [19], but the role of milk FA profiles in this context remains unexplored.

Thus, this study was conducted to test the hypothesis that dairy sheep with divergent FE would respond differently to a nutritional challenge, and this different response would be reflected in their milk FA composition. To this aim, we investigated whether there were differences in the temporal variation of the milk FA profile in dairy ewes with contrasting FE estimates and subjected to an acute underfeeding challenge.

## 2. Materials and Methods

### 2.1. Ethics Statements

All experimental procedures were approved by the Research Ethics Committees of the *Instituto de Ganadería de Montaña*, the Spanish National Research Council (CSIC), and the *Junta de Castilla y León* (Spain), following proceedings described in Spanish and European Union legislation (Royal Decree 53/2013 and Council Directive 2010/63/EU).

### 2.2. Animals, Management, and Feed Efficiency Estimation

The analyses described here are part of a larger study conducted to investigate the relationship between FE and resilience in dairy ewes. The experimental design and methodology were extensively described in a first publication that studied response/recovery profiles in animal performance (e.g., changes in dry matter intake and milk yield), ruminal fermentation parameters, and blood metabolites [19].

Briefly, the trial was conducted with 40 Assaf ewes in their first lactation (body weight = 64.8 ± 2.31 kg; days in milk = 44.3 ± 2.58; milk yield = 2.52 ± 0.171 kg/d). Sheep were housed in individual pens and fed ad libitum a total mixed ratio (TMR; 50:50) containing 198 g of crude protein, and 309 g of neutral detergent fiber/kg of dry matter (for more details, please refer to Barrio et al. [19]). Animals were milked twice daily (0830 and 1830 h) in a 1 × 10 stall milking parlor (DeLaval, Madrid, Spain).

As previously described [19], dry matter intake and milk yield and composition, as well as BW changes, were measured over a 3-week period to estimate two FE proxies: first, an FE index (FEI) was calculated as the difference between the actual and predicted intake estimated through net energy requirements for maintenance, milk production, and weight change. Second, the residual feed intake (RFI) was estimated as the residuals from a regression model that included dry matter intake, energy-corrected milk yield, metabolic body weight, and the interaction of body weight and its daily change. Using both indices allowed a more robust classification of animals, ensuring that efficiency was not biased by the limitations of a single measure. On this basis, the 9 animals with the lowest (L-FE group) and the 9 with the highest (H-FE group) FE values, for both FEI (H-FE = 0.131 ± 0.030; L-FE = 0.546 ± 0.018) and RFI (H-FE = −0.120 ± 0.024; L-FE = 0.122 ± 0.031), were selected for the nutritional challenge. This sample size was chosen to represent the extreme phenotypes while maintaining sufficient statistical power to detect biologically and statistically relevant differences, avoiding any overlap between L-FE and H-FE groups.

### 2.3. Nutritional Challenge, Sample Collection, and Processing

After FE estimation, the selected ewes, H-FE (n = 9) and L-FE (n = 9), were subjected to an acute nutritional challenge by withdrawing the TMR and feeding them only wheat straw (chopped through a 3 cm screen) for 3 days. This restriction was intended to mimic a sudden feed shortage scenario, such as one resulting from an extreme weather event or a severe disruption in the supply of raw materials. The duration was selected to induce a clear but reversible nutritional stress without compromising animal health, and to allow the evaluation of short-term metabolic and milk FA responses.

Representative samples of the TMR and the wheat straw were collected throughout the trial, stored at −30 °C, and then freeze-dried prior to FA analysis. Individual milk samples were collected (i) on 2 consecutive days before the challenge (pre-challenge period), (ii) on the last day of the challenge period, and (iii) on days 8 and 9 after the challenge (post-challenge period; when ewes were known to have fully stabilized dry matter intake and milk yield values after the challenge) [19]. Composite samples of untreated milk produced by each ewe were prepared daily according to individual yields in both morning and evening milkings and stored at −30 °C for FA determinations.

### 2.4. Laboratory Analysis

The fatty acid methyl esters (FAME) of lipids in freeze-dried samples of TMR and wheat straw (Table 1) were prepared in a 1-step extraction-transesterification procedure [20] using tridecanoic acid (Sigma-Aldrich, Saint Louis, MO, USA) as an internal standard. Separation and quantification of FAME were conducted using a gas chromatograph equipped with a flame ionization detector (GC-FID; Agilent 7890A GC System, Agilent Technologies Inc., Santa Clara, CA, USA) as explained below for milk samples. Peaks were identified based on retention time comparisons with commercially available standards.

Lipids in 1 mL of milk were extracted and converted to FAME by base-catalyzed transesterification [20]. The total FAME profile was determined by GC-FID (Agilent 7890A GC System, Santa Clara, CA, USA) using a 100 m fused silica capillary column (CP-SIL 88, Varian, Varian Ibérica S.A., Madrid, Spain) and hydrogen as fuel and carrier gas. The total FAME profile was determined using a temperature gradient program [20] and 18:1 isomers were further resolved in a separate analysis under isothermal conditions at 170 °C [20]. All peaks were identified based on retention time comparisons with commercially available FAME standards (GLC463, U-37-M, U-43-M, U-45-M and U-64-M, Nu-Chek Prep., Elysian, MN, USA; 18919-1AMP Supelco, L6031, L8404 and O5632, Sigma-Aldrich, St. Louis, MO, USA; and 11-1600-8, 20-2024-1, 20-2210-9, 20-2305-1-4, 21-1211-7, 21-1413-7, 21-1614-7, 21-1615-7 and BR mixtures 2 and 3, Larodan AB, Solna, Sweden) and cross-referencing chromatograms reported in the literature [20,21]. Comparisons were also made with reference samples for which the FA composition was determined based on GC-FID analysis of FAME and gas chromatography–mass spectrometry analysis of corresponding 4,4-dimethyloxazoline derivatives [22].

### 2.5. Statistical Analyses

Given that the milk FA compositional data did not conform to normality even after normalization with a centering and log ratio followed by a z-score, non-parametric methods were employed for statistical analysis, focusing on a factorial repeated measures design. The analysis was conducted using R software [23].

Data were subjected to a factorial analysis using the *nparLD* package [24], which provides Wald-type test statistics to evaluate the main effects of group (H-FE vs. L-FE) and period (pre-challenge, challenge, and post-challenge), and their interaction. Least square means were compared using the *lsmeans* function [25]. Post hoc comparisons between time points and the interaction between efficiency and time were performed using the *nparcomp* package to estimate pairwise differences. *p*-values were always adjusted using a 5% False Discovery Rate (FDR) method. Therefore, FDR-adjusted *p*-values (abbreviated as FDR) are always reported. Differences were declared significant at FDR < 0.05.

Principal component analysis (PCA) was performed to explore the variance in FA data across the FE groups and the experimental periods. This analysis was conducted on the z-score data using the *prcomp* function in R software. The first two principal components (PC1 and PC2) were extracted, and the variance explained by each component was calculated. To visualize the results of the PCA, a scatter plot was generated using *ggplot2* [26]. Additionally, the contribution of each FA to the PC1 was evaluated using the following formula:

$$C_{ij}=\;{\left(L\right)}_{ij}^{2}\;\times \;{Var}_{j}$$

where C*_ij_* represents the contribution of the *i*-th FA to the *j*-th PC, L*_ij_* is the loading of the *i*-th variable on the *j*-th PC, and Var*_j_* is the proportion of total variance explained by the *j*-th PC.

To identify the most influential FA, their contributions to the PC1 were calculated and plotted in descending order. The elbow rule was then applied by examining the contribution plot for an inflection point, where the slope of the curve decreases significantly. The FAs located to the left of this point, showing a sharp decline in contribution afterwards, were considered the most relevant, as they explain the highest percentage of the variation observed in the PC1, and were retained for further analysis. Then, a second PCA was performed with this subset of FA to assess whether they effectively capture the main sources of variation and to evaluate their discriminatory potential.

In addition to the unsupervised PCA, a supervised method was also used. A sparse partial least squares discriminant analysis (sPLS-DA) was performed for classification according to FE (H-FE vs. L-FE) while simultaneously selecting the most discriminative variables. This supervised model was fitted using the *mixOmics* package [27]. The optimal number of components and the number of variables retained per component were determined through cross-validation, minimizing the classification error rate.

## 3. Results

### 3.1. Milk Fatty Acid Profile

As shown in Table 2, in all cases, when a significant interaction of group × period was initially observed (FDR < 0.05), it disappeared for pairwise comparisons (FDR > 0.05).

Regarding differences between both FE groups, milk FA composition only slightly differed. Significantly higher levels of *iso* 17:0, *anteiso* 17:0, and 18:0 were found in the L-FE group (FDR < 0.05).

In contrast, most of the FAs were affected by the period (FDR < 0.05). Thus, most short- and medium-chain FAs (i.e., FA with ≤16 carbons), except for 16-carbon monounsaturated FA (MUFA), decreased their concentrations in milk during the challenge (FDR < 0.05). Exceptions included 4:0, 5:0, 6:0, and *anteiso* 13:0, which showed no changes (FDR > 0.05). After the challenge, all affected FAs returned to their initial concentrations, but those of 9:0, 11:0, 14:0, and 15:0 were even higher (FDR < 0.001). Regarding longer FAs, 13-oxo-18:0, 17:0, 18:0, and most of the 16–18-carbon MUFAs increased during the challenge, whereas those of *cis*-16 18:1, *trans*-11 18:1, and *trans*-13 + 14 18:1 decreased (FDR < 0.01) and *trans*-15 18:1 showed similar values in both periods (FDR > 0.05). Some of these FAs, such as *anteiso* 17:0, *cis*-9, *cis*-11, and *cis*-12 isomers of 18:1, did not recover their initial concentrations after the challenge (FDR < 0.001).

As shown in Table 2, whereas *cis*-9, *trans*-12 and *trans*-9, *cis*-12 18:2, and the summation of other *trans*, *trans* conjugated linoleic acids (CLA) were similar in the pre-challenge and challenge periods (FDR > 0.05), whereas the remaining 18:2 isomers showed their highest concentrations during the challenge (FDR < 0.001). In the post-challenge, all of them recovered their initial values, except *trans*-10, *cis*-12 CLA (FDR > 0.05). The concentrations of 18:3n-6, 18:3n-3, most saturated FAs (SFAs) with 20–24 carbons (i.e., 20:0, 22:0, 23:0, and 24:0), *cis*-13 22:1, and 22:5n-3 decreased with the feed restriction, whereas 19:0, *cis*-9 20:1, *cis*-13 20:1, 20:2n-6, 20:3n-6, 21:0, and *cis*-15 24:1 increased (FDR < 0.05).

As shown in Table 3, none of the summations of milk FA differed significantly between H-FE and L-FE (FDR > 0.05), but most of them were affected by the period (FDR < 0.001). Thus, the sum of <C16, C16, n-3 polyunsaturated FA (PUFA) C20-22, n-3 PUFA, even-chain de novo SFA, and the ratio of even-chain de novo SFA/*cis*-9 18:1 decreased during the challenge (FDR < 0.05). In contrast, the summation of >C16 FA, MUFA, PUFA, n-6 PUFA, odd-chain FA (OCFA), *cis* and *trans* 18:1 isomers, non-conjugated 18:2, and CLA showed higher values in the challenge (FDR < 0.001). In the post-challenge, the summation of >C16, n-3 PUFA C20-22, and *cis* 18:1 reached the lowest concentrations, the total SFA and the even-chain de novo SFA/*cis*-9 18:1 ratio were the highest (FDR < 0.05), and the others showed similar values compared to the pre-challenge period (FDR > 0.05).

**Table 3:** Summations of the milk fatty acid (FA) profile in the high-feed efficiency (H-FE) and low-feed efficiency (L-FE) groups of ewes subjected to an acute nutritional challenge (i.e., fed only straw for 3 d). Measurements and samplings were conducted (i) before the challenge (Pre), (ii) at the end of the challenge (Challenge), and (iii) 8–9 d after the challenge (Post).

	Group		Period					FDR ^2^	
Item, g/100 g of FA	H-FE	L-FE	Pre	Challenge	Post	SED ^1^	Group	Period	G × P
Σ <C16	28.77	27.94	36.10 ^a^	10.13 ^b^	38.83 ^a^	3.453	0.826	<0.001	0.993
Σ C16	29.21	28.19	32.75 ^a^	20.02 ^b^	33.32 ^a^	1.425	0.457	<0.001	0.417
Σ >C16	41.89	43.73	31.04 ^b^	69.65 ^a^	27.75 ^c^	4.616	0.204	<0.001	0.172
Σ SFA	67.31	66.47	76.74 ^b^	45.40 ^c^	78.53 ^a^	3.674	0.826	<0.001	0.501
Σ MUFA	27.48	28.19	18.59 ^b^	47.72 ^a^	17.21 ^b^	2.583	0.724	<0.001	0.417
Σ PUFA	5.30	5.40	4.90 ^b^	6.64 ^a^	4.50 ^b^	0.379	0.770	<0.001	0.501
Σ n-3 PUFA C20-22	0.165	0.183	0.182 ^a^	0.178 ^b^	0.161 ^c^	0.0246	0.319	0.012	0.585
Σ n-3 PUFA	0.762	0.829	0.885 ^a^	0.719 ^b^	0.781 ^ab^	0.0673	0.315	<0.001	0.835
Σ n-6 PUFA C20-22	0.302	0.329	0.302	0.352	0.292	0.0416	0.411	0.312	0.005 *
Σ n-6 PUFA	3.27	3.31	2.90 ^b^	4.35 ^a^	2.63 ^b^	0.307	0.826	<0.001	0.379
Σ odd-chain FA	2.89	2.99	2.43 ^b^	3.85 ^a^	2.55 ^b^	0.289	0.796	<0.001	0.821
Σ branched-chain FA	2.03	2.11	2.10	2.03	2.08	0.107	0.238	0.474	0.457
Even-chain de novo SFA	26.36	25.57	33.25 ^a^	9.01 ^b^	35.64 ^a^	3.206	0.879	<0.001	0.991
Even-chain de novo SFA/*cis*-9 18:1	2.08	1.92	2.62 ^b^	0.32 ^c^	3.07 ^a^	0.249	0.415	<0.001	0.557
Σ *cis* 18:1	21.57	22.48	13.73 ^b^	39.95 ^a^	12.39 ^c^	2.355	0.320	<0.001	0.227
Σ *trans* 18:1	2.61	2.51	1.95 ^b^	3.95 ^a^	1.77 ^b^	0.522	0.749	<0.001	0.696
Σ non-conjugated 18:2	3.59	3.61	3.11 ^b^	4.89 ^a^	2.79 ^b^	0.326	0.909	<0.001	0.470
Σ CLA	0.435	0.417	0.397 ^b^	0.463 ^a^	0.417 ^ab^	0.0241	0.555	<0.001	0.375

^1^ SED = standard error of the difference. ^2^ *p*-values adjusted using a 5% False Discovery Rate (FDR). * No significant differences (FDR > 0.05) in the interaction G × P were found for pairwise comparisons. ^a–c^ Within a row, different superscripts indicate significant differences (FDR < 0.05).

### 3.2. Clustering

**Figure 1:**
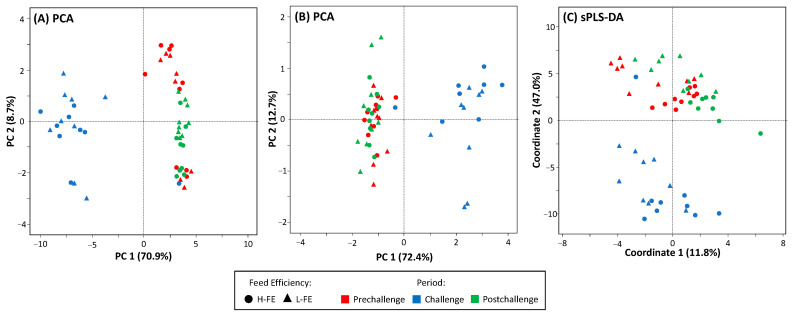
(**A**) Principal component analysis (PCA) of the complete milk fatty acid data; (**B**) PCA of a subset of the 10 milk fatty acids contributing the most to the variation explained by the PC1; (**C**) sparse partial least squares discriminant analysis (sPLS-DA) of the complete milk fatty acid data. Data include the pre-challenge (red), challenge (blue), and post-challenge (green) periods for high feed efficiency (H-FE; circles) and low feed efficiency (L-FE; triangles) dairy ewes.

**Figure 2:**
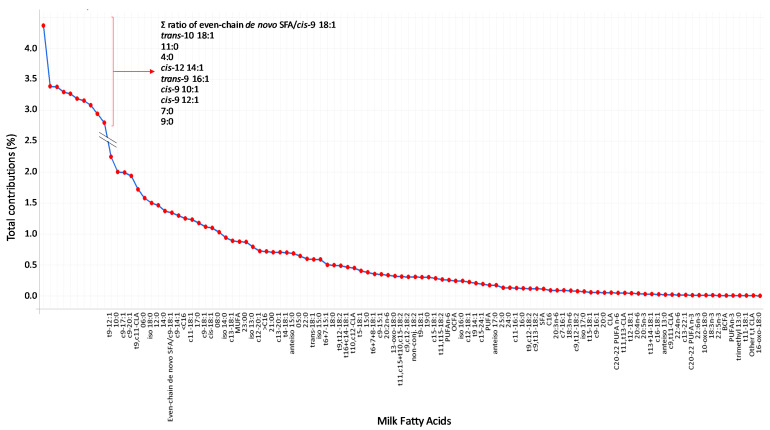
Contribution of each fatty acid (FA) to the first principal component (PC1) obtained from the PC analysis, including all milk FA data through the experiment (i.e., in the pre-challenge, challenge, and post-challenge periods). The dots represent the relative contribution (%) of each FA in order of magnitude to the PC1. The red bracket highlights the 10 FA selected by the elbow rule, which together explain more than 30% of the variation.

Figure 1A shows the score plot of the PCA performed with the milk FA profile data. The PC1 explained 70.9% of the variability, and PC2 only accounted for 8.7%. The FE groups were not separated, but the PCs clearly clustered the periods: one cluster for the challenge data and another for both pre- and post-challenge data. This clustering affected all ewes but one whose challenge and pre- and post-challenge data overlapped. Figure 2 shows the total contributions of FA to PC1. Those contributing more than 3% each, more than 30% all together, to the explained variation of PC1 were retained as the most influential in driving the variation, as they exceeded the threshold identified by the elbow rule. These were Σ ratio of even-chain de novo SFA/*cis*-9 18:1, *trans*-10 18:1, 11:0, 4:0, *cis*-12 14:1, *trans*-9 16:1, *cis*-9 10:1, *cis*-9 12:1, 7:0, and 9:0, in discerning order.

Figure 1B shows the second PCA, performed with the 10 selected FAs. In this case, PC1 and PC2 explained 72.4% and 12.7% of the variability, respectively, maintaining the challenge and the combined pre- and post-challenge clusters. As in the first PCA, H-FE and L-FE were not separated and the same ewe showed overlapping data across periods.

Figure 1C shows the score plot of the sPLS-DA. The first coordinate explained 11.8% of the total variance and was the axis that separated L-FE and H-FE, although this separation remained limited. In contrast, the second coordinate, which explained 47.0% of the variance, clearly discriminated between two clusters: one for the challenge and one for the pre- and post-challenge periods. Notably, the same ewe whose challenge sample overlapped with the pre- and post-challenge cluster in the PCA remained within this cluster in the sPLS-DA, confirming its consistent behavior across both analyses.

## 4. Discussion

Feed efficiency is a key factor in enhancing the sustainability of dairy production [13,14,15], and, although insufficiently explored, the milk FA profile has been proposed as a potential non-invasive indicator of this trait [3,4]. Moreover, previous findings in Assaf ewes suggest that divergent FE might be associated with differences in nutrient partitioning [3,19,28]. Therefore, investigating whether milk FA composition can reflect FE becomes particularly relevant in the context of underfeeding, as it may reveal mechanisms by which more or less efficient animals are able to cope with nutritional stress.

### 4.1. Feed Efficiency and Milk Fatty Acid Composition

To facilitate the interpretation and discussion of the results of this study, it may be useful to recall that more efficient animals (H-FE) were more productive at similar dry matter intake. Thus, consumption values (2.97 vs. 2.85 kg of a total mixed ration/d in the pre-challenge) did not significantly vary between groups throughout the trial. On the other hand, milk yield was higher in H-FE than in L-FE (2.847 vs. 2.133 kg/d in the pre-challenge period), and this group (H-FE) showed a quicker response to and recovery from the feed restriction than L-FE, as already reported and discussed in Barrio et al. [19].

Consistent with their higher milk production, a greater milk fat yield was observed in H-FE than in L-FE ewes (126.7 vs. 89.3 g/day; Barrio et al. [19]). However, despite this variation in fat yield, the current analysis of milk FA composition revealed only minor differences between L- and H-FE. For example, no significant variations were found in total branched-chain FAs, but the proportions of *iso* 17:0 and *anteiso* 17:0 were higher in L-FE ewes. These FAs, which might be derived from post-ruminal metabolism (by elongation) but are mainly produced by microbial metabolism in the rumen, may reflect potential differences in fermentation patterns associated with FE [29,30]. This is in line with the higher total VFA production previously reported in the L-FE [19].

Preformed FA in milk may be partially derived from plasma non-esterified fatty acids (NEFA), which are directly linked to adipose tissue mobilization during lactation [1,31]. However, no significant changes in plasma NEFA were detected between more and less efficient ewes in our previous study [19], which would rule out the explanation that the higher level of 18:0, one of the main components of NEFA, in L-FE animals was due to increased mobilization [32,33]. Additionally, as 18:0 represents the final product of ruminal biohydrogenation, its greater values could reflect a greater extent of this process, which seems contrary to the findings [3] in Assaf sheep divergent for FE. A possible explanation for discrepancies between the present study and the previously reported study [3] is that the former involved primiparous ewes, which may differ in terms of lipid mobilization and deposition dynamics from the multiparous animals used in the latter. Therefore, rather than patterns directly associated with FE, these findings may indicate substantial individual variability and the influence of parity on lipid metabolism and tissue mobilization, which warrants further research.

The few differences observed in some individual milk FAs were not mirrored in changes in the FA summations (Table 3), reinforcing that milk FA composition was only slightly affected by FE. In this respect, a previous study [3] reported that differences in the FA profiles between high- and low-feed efficiency sheep were more pronounced in the rumen content than in milk, where they were present but less evident. Overall, the results suggest that, despite differences in certain individual FAs (i.e., *iso* and *anteiso* 17:0, and 18:0) pointing to the previously discussed subtle variations in lipid metabolism (either in the rumen or post-ruminally), processes driving feed efficiency would barely alter the relative abundance of specific FAs in the present study.

This was supported by PCA, where the use of the complete FA profile did not allow for a differential clustering of L- and H-FE groups. Neither the selection of the 10 FAs contributing over 30% of the variability, which aimed to reduce the influence of less informative variables, nor the supervised sPLS-DA allow for a clear discrimination. In contrast, the challenge period data were clearly separated from the rest of the data (i.e., pre- and post-challenge data), highlighting a much stronger influence of the feed restriction on the milk FA profile. It is worth noting that, given the weight of the challenge, analyses to discriminate by FE were also performed using only the pre-challenge or pre-challenge + post-challenge data, but in neither case was it possible to separate L-FE and H-FE.

### 4.2. Impact of the Nutritional Challenge on Milk Fatty Acid Composition

Nutritional challenges are known to impact lipid metabolism in dairy ruminants, leading to shifts in the milk FA profile [1,10]. During periods of underfeeding, animals increase body fat mobilization, which alters the proportions of de novo synthesized and preformed FA in milk [34,35]. These changes provide insight into metabolic adaptation to nutrient fluctuations, making the milk FA profile a potential indicator of physiological responses to nutritional stress.

The marked reduction in most short- and medium-chain FAs (≤C16, except for 16-carbon MUFA) during the challenge indicates a decrease in de novo FA synthesis in the mammary gland. This pattern may be explained by feed restriction causing a reduction in the availability of VFA [19,28], the main substrates for de novo synthesis in ruminants [7,9].

A transition from de novo synthesis to mobilization of body fat [1,36] was reflected by the sharp decrease in the ratio of even-chain de novo SFA/*cis*-9 18:1 and the higher percentage of certain long-chain FAs (e.g., 18:0, 19:0, 21:0, and most MUFAs with 16–18 carbons). Similar patterns were observed in the milk of dairy goats fed 70% of their energy and protein requirements [34]. Furthermore, the reduction in feed supply during the challenge would have affected ruminal microbiota and therefore biohydrogenation, leading to shifts in *trans*-FA intermediates [37,38]. This resulted in a greater concentration of *trans*-10 18:1 at the expense of *trans*-11 and *trans*-13 + 14 isomers, which could have further contributed to the reduction in de novo FA synthesis [3,22,39]. Under nutritional restriction, this reduction might allow the limited pool of VFA to be redirected, under nutritional restriction, toward metabolic processes that are more critical for the animal’s survival [40,41].

Short OCFAs (i.e., with 15 or fewer carbons) can be synthesized de novo in the mammary gland from propionate, whereas longer OCFAs (≥C17) might be more influenced by microbial activity and dietary sources [29,42,43]. Although their total summation increased during feed restriction, this was mainly driven by the rise in long OCFAs, likely due to increased uptake [2]. In contrast, short OCFAs showed a decrease, supporting the idea of a metabolic shift under feed restriction that favors uptake over de novo synthesis in the mammary gland [1,11]. This shift might also be related to changes in the rumen caused by the severe restriction, likely disrupting the ruminal fermentation [7,9]. Alterations such as reduced ammonia levels and VFA production and increased pH [19] would have affected ruminal lipid metabolism, influencing not only the digesta FA profile but also, consequently, the milk FA composition. The relative increase caused by the challenge in C17 FA, including both linear (17:0 and *cis*-9 17:1) and branched forms (*iso* and *anteiso* 17:0), points to a contribution of both ruminal and post-ruminal processes. In fact, although these odd- and branched-chain FAs are derived mainly from bacteria leaving the rumen, they can also be derived from de novo synthesis and desaturation and elongation processes in the mammary gland, and from adipose tissue mobilization [29,44].

Regarding PUFAs, most of them increased with the nutritional challenge. This shift may be explained by the compositional nature of the milk FA data: the lower the proportion of de novo FA, the higher the proportion of preformed FA. In addition, the contribution of tissue mobilization during feed restriction to this rise in PUFAs should not be ignored [1,41]. Nevertheless, despite this overall trend, 18:3n-3 and 18:3n-6, which are strongly dependent on dietary intake, decreased. In contrast, 18:2n-6 and its elongation products (e.g., 20:2n-6) increased, possibly due to enhanced body fat mobilization, which may have served as a substrate for hepatic synthesis of C20–22 n-6 PUFA [45,46].

Other long-chain FAs (>C18) showed variable dynamics during the feed restriction. Some increased (e.g., *cis*-9, *cis*-13 20:1, *cis*-15 24:1) possibly due to a combination of tissue mobilization and desaturase activity in the mammary gland to maintain the melting point [1,32]. Some others decreased (e.g., 20:0, 22:0, 23:0, 24:0), likely due to their reduced intake [34,35]. Nevertheless, the explanation for the lower concentration of 22:5n-3 is not that simple when compared to the stability of 20:5n-3 and 22:6n-3 observed during the challenge, which suggests the involvement of different regulatory mechanisms [38,47,48]. These divergent dynamics of different long-chain PUFAs might be accounted for by their diet availability or a selective uptake of specific plasma FAs by the mammary gland [49]. In the absence of lipid supply from the diet, the mammary gland might preferentially incorporate FAs derived from body fat depots [1,2]. However, if these incorporations were the predominant mechanism, a generalized increase in non-dietary FAs would be expected, which was not observed in this study.

Finally, during the post-challenge period, most FAs returned to initial values, as confirmed by PCA and sPLS-DA, which always clustered both periods together. Incidentally, it should be noted that one H-FE animal did not exhibit a clear separation during the challenge, indicating that its FA profile was not significantly affected by the underfeeding. Since this concerns only one animal, its analysis as an example of resilience would be of limited value. Nevertheless, further research on the issue of individual variability is warranted.

Only three FAs did not recover their pre-challenge concentrations: the summations of FAs with more than 16 carbons and of *cis* 18:1 and n-3 PUFA C20-22, which could be attributed to reduced tissue mobilization upon refeeding. This is consistent with the decreased NEFA content observed previously in the post-challenge period [19]. The lower proportion of preformed FA was in line with the rise in the ratio of even-chain de novo SFA/*cis*-9 18:1 and in total SFA during the post-challenge. Regarding the latter, the summation of SFA was largely driven by the increase in individual SFA originating de novo in the mammary gland [10,41,44], such as 4:0, 14:0, and 15:0, which greatly exceeded their initial values.

Overall, most observed changes in milk FA composition would reflect adaptive responses by dairy ewes to the acute nutritional challenge, regardless of their divergent FE. Nevertheless, findings are specific to the experimental conditions and to the Assaf breed, which may limit their applicability to other contexts.

## 5. Conclusions

The limited differences in the milk FA profile between dairy ewes with a higher (H-FE) or lower (L-FE) feed efficiency and subjected to an acute nutritional challenge (i.e., 3 days receiving only straw) suggest that FE would have a minor impact on the relative abundance of specific FAs in milk. Despite some variations in a few metabolites (i.e., *iso* and *anteiso* 17:0, and 18:0), which would warrant further research, the milk FA composition might not be a suitable marker for clustering more and less efficient animals under this scenario. On the other hand, and although this was not the main aim of this study, shifts in the balance between de novo and preformed FA and in certain specific FAs (e.g., some OCFAs, C17 FA, or very long-chain FAs) in response to the nutritional challenge prove that the milk FA profile is very sensitive to underfeeding, and point to the involvement of different regulatory mechanisms (including body fat mobilization, altered ruminal biohydrogenation, and post-ruminal lipid metabolism). Overall, these findings support the relevance of milk FA analysis for understanding the response to nutritional stress.

## Figures and Tables

**Table 1:** Fatty acid (FA) composition of the total mixed ration (TMR) and the wheat straw.

	TMR	Wheat Straw
Total FA, g/100 g of DM	1.90	0.67
FA profile, g/100 g of total FA		
12:0	0.47	1.34
14:0	0.83	4.38
15:0	0.47	0.87
16:0	23.48	34.88
*cis*-9 16:1	0.24	0.65
17:0	0.41	0.61
18:0	4.13	4.72
*cis*-9 18:1	12.92	11.14
*cis*-11 18:1	0.86	0.85
*cis*-9, *cis*-12 18:2	35.31	12.74
20:0	1.21	4.71
18:3n-3	13.28	2.78
*cis*-11 20:1	0.35	0.54
22:0	1.21	3.43
24:0	1.49	2.75

**Table 2:** Milk fatty acid (FA) profile in the high-feed efficiency (H-FE) and low-feed efficiency (L-FE) ewes subjected to an acute nutritional challenge (i.e., fed only straw for 3 d). Measurements and samplings were conducted (i) before the challenge (Pre), (ii) at the end of the challenge (Challenge), and (iii) 9–10 d after the challenge (Post).

	Group		Period					FDR ^2^	
Item, g/100 g of FA	H-FE	L-FE	Pre	Challenge	Post	SED ^1^	Group	Period	G × P
4:0	1.48	1.17	1.18 ^ab^	0.80 ^b^	1.99 ^a^	0.650	0.480	<0.001	0.838
5:0	0.009	0.007	0.009 ^ab^	0.004 ^b^	0.011 ^a^	0.0030	0.084	<0.001	0.796
6:0	1.44	1.18	1.39 ^ab^	0.67 ^b^	1.86 ^a^	0.535	0.504	<0.001	0.909
7:0	0.038	0.027	0.038 ^a^	0.016 ^b^	0.043 ^a^	0.0155	0.368	0.001	0.749
8:0	1.98	1.92	2.45 ^a^	0.81 ^b^	2.59 ^a^	0.271	0.943	<0.001	0.891
9:0	0.047	0.043	0.053 ^b^	0.009 ^c^	0.072 ^a^	0.0064	0.183	<0.001	0.941
10:0	7.25	7.30	9.89 ^a^	2.09 ^b^	9.84 ^a^	0.922	0.606	<0.001	0.498
11:0	0.073	0.070	0.085 ^b^	0.012 ^c^	0.117 ^a^	0.0121	0.920	<0.001	0.749
*cis-*9 10:1	0.230	0.213	0.293 ^a^	0.053 ^b^	0.319 ^a^	0.0368	0.838	<0.001	0.879
12:0	4.44	4.55	5.97 ^a^	1.45 ^b^	6.07 ^a^	0.477	0.619	<0.001	0.212
*cis-*9 12:1	0.075	0.076	0.102 ^a^	0.017 ^b^	0.107 ^a^	0.0085	0.942	<0.001	0.835
*trans-*9 12:1	0.039	0.037	0.049 ^a^	0.010 ^b^	0.056 ^a^	0.0067	0.909	<0.001	0.991
*iso* 13:0	0.020	0.020	0.027 ^a^	0.009 ^b^	0.025 ^a^	0.0028	0.943	<0.001	0.898
*anteiso* 13:0	0.010	0.010	0.010 ^ab^	0.008 ^b^	0.012 ^a^	0.0016	0.879	0.002	0.991
4,8,12 trimethyl 13:0	0.051	0.052	0.055 ^a^	0.045 ^b^	0.054 ^a^	0.0049	0.851	0.004	0.879
14:0	9.77	9.45	12.37 ^b^	3.18 ^c^	13.28 ^a^	1.036	0.844	<0.001	0.944
*iso* 14:0	0.093	0.101	0.119 ^a^	0.041 ^b^	0.131 ^a^	0.0128	0.501	<0.001	0.501
*cis-*9 14:1	0.161	0.142	0.180 ^a^	0.053 ^b^	0.221 ^a^	0.0239	0.667	<0.001	0.749
*cis-*12 14:1	0.061	0.064	0.083 ^a^	0.013 ^b^	0.091 ^a^	0.0125	0.911	<0.001	0.392
*trans-*9 14:1	0.010	0.009	0.008	0.012	0.008	0.0032	0.679	0.462	0.826
15:0	0.818	0.815	0.928 ^b^	0.432 ^c^	1.090 ^a^	0.062	0.821	<0.001	0.906
*iso* 15:0	0.185	0.192	0.245 ^a^	0.093 ^b^	0.226 ^a^	0.0251	0.368	<0.001	0.249
*anteiso* 15:0	0.347	0.355	0.428 ^a^	0.161 ^b^	0.464 ^a^	0.0417	0.813	<0.001	0.737
*cis-*9 15:1	0.010	0.009	0.008	0.009	0.010	0.0025	0.457	0.341	0.515
*trans-*6 + 7 15:1	0.013	0.013	0.016 ^a^	0.007 ^b^	0.017 ^a^	0.0024	0.991	<0.001	0.501
16:0	27.23	26.27	30.93 ^a^	17.89 ^b^	31.43 ^a^	1.419	0.462	<0.001	0.411
*iso* 16:0	0.249	0.263	0.301 ^a^	0.156 ^b^	0.311 ^a^	0.0333	0.341	<0.001	0.707
*cis-*7 16:1	0.316	0.299	0.294 ^b^	0.349 ^a^	0.279 ^b^	0.0269	0.770	<0.001	0.470
*cis-*9 16:1	0.847	0.812	0.769 ^b^	0.906 ^a^	0.813 ^ab^	0.0800	0.796	<0.001	0.588
*cis-*11 16:1	0.015	0.015	0.013 ^b^	0.019 ^a^	0.014 ^b^	0.0021	0.709	0.003	0.838
*trans-*9 16:1	0.047	0.059	0.016 ^b^	0.125 ^a^	0.018 ^b^	0.0240	0.944	<0.001	0.080
17:0	1.05	1.12	0.67 ^b^	1.99 ^a^	0.61 ^b^	0.165	0.404	<0.001	0.562
*iso* 17:0	0.320	0.343	0.338 ^a^	0.366 ^a^	0.290 ^b^	0.0144	0.034	<0.001	0.879
*anteiso* 17:0	0.499	0.534	0.473 ^b^	0.643 ^a^	0.433 ^c^	0.0305	0.038	<0.001	0.784
*cis-*9 17:1	0.521	0.564	0.247 ^b^	1.143 ^a^	0.238 ^b^	0.1383	0.991	<0.001	0.320
18:0	8.68	9.45	7.51 ^b^	13.38 ^a^	6.30 ^c^	0.881	0.019	<0.001	0.462
*iso* 18:0	0.116	0.119	0.058 ^b^	0.227 ^a^	0.068 ^b^	0.0176	0.501	<0.001	0.744
10-oxo-18:0 ^3^	0.015	0.016	0.016	0.015	0.017	0.0065	0.573	0.747	0.844
13-oxo-18:0	0.021	0.021	0.015 ^b^	0.031 ^a^	0.019 ^ab^	0.0085	0.977	0.004	0.796
16-oxo-18:0	0.017	0.015	0.017	0.015	0.017	0.0031	0.770	0.417	0.657
*cis*-9 18:1	20.45	21.32	12.91 ^b^	38.06 ^a^	11.69 ^c^	3.133	0.375	<0.001	0.152
*cis-*11 18:1	0.708	0.743	0.446 ^b^	1.351 ^a^	0.379 ^c^	0.1256	0.737	<0.001	0.805
*cis-*12 18:1	0.223	0.228	0.218 ^b^	0.285 ^a^	0.172 ^c^	0.0171	0.909	<0.001	0.561
*cis-*13 18:1	0.065	0.061	0.042 ^b^	0.105 ^a^	0.042 ^b^	0.0115	0.212	<0.001	0.107
*cis-*15 18:1	0.084	0.079	0.069 ^b^	0.110 ^a^	0.066 ^b^	0.0098	0.298	<0.001	0.399
*cis-*16 18:1	0.041	0.045	0.049 ^a^	0.039 ^b^	0.041 ^ab^	0.0041	0.460	<0.001	0.879
*trans-*4 18:1	0.018	0.020	0.011 ^b^	0.032 ^a^	0.014 ^b^	0.0077	0.249	<0.001	0.550
*trans-*5 18:1	0.013	0.013	0.010 ^b^	0.018 ^a^	0.011 ^b^	0.0024	0.826	<0.001	0.895
*trans-*6 + 7 + 8 18:1	0.199	0.203	0.177 ^b^	0.278 ^a^	0.149 ^b^	0.0301	0.737	<0.001	0.460
*trans-*9 18:1	0.169	0.153	0.131 ^b^	0.221 ^a^	0.131 ^b^	0.0173	0.823	<0.001	0.779
*trans-*10 18:1	1.004	0.906	0.315 ^b^	2.278 ^a^	0.272 ^b^	0.4725	0.557	<0.001	0.196
*trans-*11 18:1	0.569	0.550	0.596 ^a^	0.472 ^b^	0.611 ^a^	0.0499	0.667	0.002	0.679
*trans-*12 18:1	0.201	0.200	0.206	0.210	0.185	0.0176	0.942	0.085	0.914
*trans-*13 + 14 18:1	0.172	0.170	0.180 ^a^	0.101 ^b^	0.232 ^a^	0.0325	0.858	<0.001	0.147
*trans-*15 18:1	0.260	0.295	0.324 ^a^	0.342 ^ab^	0.167 ^b^	0.1262	0.749	<0.001	0.508
*trans*-16 + *cis*-14 18:1 ^4^	0.38	0.34	0.28 ^b^	0.54 ^a^	0.25 ^b^	0.110	0.991	<0.001	0.017 *
*cis-*9, *cis-*12 18:2 ^5^	2.89	2.90	2.50 ^b^	3.93 ^a^	2.23 ^b^	0.284	0.895	<0.001	0.614
*cis-*9, *trans-*12 18:2 ^6^	0.084	0.084	0.083 ^ab^	0.095 ^a^	0.076 ^b^	0.0076	0.838	<0.001	0.020 *
*cis-*9, *trans-*13 18:2 ^6^	0.150	0.155	0.148 ^b^	0.180 ^a^	0.131 ^b^	0.0117	0.891	<0.001	0.050
*trans-*9, *cis-*12 18:2	0.026	0.027	0.025 ^ab^	0.030 ^a^	0.023 ^b^	0.0025	0.724	<0.001	0.858
*trans-*11, *cis-*15 18:2 ^7^	0.071	0.067	0.059 ^b^	0.095 ^a^	0.053 ^b^	0.0191	0.462	<0.001	0.001 *
*trans-*9, *trans-*12 18:2	0.013	0.012	0.010 ^b^	0.018 ^a^	0.009 ^b^	0.0045	0.913	<0.001	0.262
*trans-*11, *trans-*15 18:2 ^6^	0.036	0.037	0.034 ^b^	0.047 ^a^	0.028 ^b^	0.0052	0.929	<0.001	0.562
*cis-*9, *trans-*11 CLA ^8^	0.324	0.311	0.307	0.324	0.320	0.0212	0.659	0.416	0.313
*trans-*9, *cis-*11 CLA	0.034	0.034	0.016 ^b^	0.069 ^a^	0.017 ^b^	0.0512	0.172	<0.001	0.020 *
*trans-*10, *cis-*12 CLA	0.007	0.007	0.004 ^b^	0.010 ^a^	0.007 ^a^	0.0017	0.485	<0.001	0.991
*trans-*11, *trans-*13 CLA	0.008	0.007	0.009	0.007	0.007	0.0017	0.470	0.223	0.405
Σ other *trans, trans* CLA ^9^	0.053	0.052	0.055 ^ab^	0.045 ^b^	0.057 ^a^	0.0050	0.977	0.001	0.036 *
18:3n-6	0.087	0.087	0.090 ^a^	0.065 ^b^	0.105 ^a^	0.0107	0.991	<0.001	0.096
18:3n-3 ^10^	0.597	0.646	0.703 ^a^	0.541 ^b^	0.620 ^ab^	0.0524	0.220	<0.001	0.898
19:0	0.104	0.107	0.102 ^b^	0.132 ^a^	0.082 ^b^	0.0149	0.770	<0.001	0.472
20:0	0.181	0.189	0.216 ^a^	0.143 ^c^	0.196 ^b^	0.0164	0.185	<0.001	0.067
*cis-*9 20:1	0.008	0.009	0.006 ^b^	0.014 ^a^	0.005 ^b^	0.0034	0.737	<0.001	0.469
*cis-*12 20:1	0.006	0.008	0.007	0.008	0.007	0.0019	0.175	0.845	0.737
*cis-*13 20:1	0.006	0.006	0.005 ^b^	0.009 ^a^	0.004 ^b^	0.0014	0.993	<0.001	0.844
20:2n-6	0.022	0.022	0.020 ^b^	0.029 ^a^	0.017 ^c^	0.0030	0.709	<0.001	0.845
20:3n-6	0.030	0.034	0.033 ^a^	0.037 ^a^	0.026 ^b^	0.0045	0.282	<0.001	0.006 *
20:4n-6 ^11^	0.217	0.237	0.213	0.250	0.218	0.0274	0.434	0.228	0.012 *
20:5n-3	0.048	0.049	0.052 ^a^	0.052 ^ab^	0.041 ^b^	0.0063	0.993	<0.001	0.248
21:0	0.052	0.054	0.067 ^b^	0.024 ^a^	0.068 ^b^	0.0100	0.749	<0.001	0.465
22:0	0.081	0.085	0.105 ^a^	0.041 ^b^	0.104 ^a^	0.0106	0.312	<0.001	0.227
*cis-*13 22:1	0.021	0.021	0.017 ^b^	0.019 ^b^	0.027 ^a^	0.0029	0.879	<0.001	0.127
22:4n-6	0.033	0.036	0.036	0.036	0.032	0.0043	0.401	0.022 *	0.991
22:5n-3	0.097	0.108	0.108 ^a^	0.103 ^b^	0.096 ^c^	0.0153	0.465	0.036	0.797
22:6n-3	0.021	0.026	0.023	0.024	0.024	0.0034	0.142	0.581	0.749
23:0	0.046	0.051	0.065 ^a^	0.022 ^b^	0.060 ^a^	0.0079	0.347	<0.001	0.462
24:0	0.044	0.044	0.051 ^a^	0.032 ^b^	0.050 ^a^	0.0065	0.544	<0.001	0.127
*cis-*15 24:1	0.035	0.033	0.026 ^c^	0.039 ^a^	0.036 ^b^	0.0062	0.614	0.004	0.015 *
25:0	0.013	0.014	0.014	0.012	0.015	0.0045	0.845	0.057	0.986

^1^ SED = standard error of the difference. ^2^ *p*-values adjusted using a 5% False Discovery Rate (FDR). ^3^ Coelutes with 10-OH-18:0. ^4^ Coelutes with *trans*-10, *trans*-14 18:2. ^5^ Contains *cis*-9, *cis*-15 18:2 as a minor component. ^6^ Coelutes with other 18:2 of indeterminate double bond position. ^7^ Coelutes with *trans*-10, *cis*-15 18:2. ^8^ Contains *trans*-7, *cis*-9 and *trans*-8, *cis*-10 CLA as minor components. ^9^ Sum of *trans*-8, *trans*-10, *trans*-9, *trans*-11, *trans*-10, *trans*-12 CLA. ^10^ Coelutes with *cis*-11 20:1. ^11^ Coelutes with an unidentified compound. ^a–c^ Within a row, different superscripts indicate significant differences (FDR < 0.05). * No significant differences (FDR > 0.05) in the interaction G × P were found for pairwise comparisons.

## Data Availability

The original contributions presented in this study are included in the article. Further inquiries can be directed to the corresponding author.

## Abbreviations

The following abbreviations are used in this manuscript:

CLA	Conjugated Linoleic Acid
FAs	Fatty Acids
FAMEs	Fatty Acid Methyl Esters
FDR	False Discovery Rate
FE	Feed Efficiency
FEI	Feed Efficiency Index
GC-FID	Gas Chromatograph Equipped with a Flame Ionization Detector
H-FE	High Feed Efficiency
L-FE	Low Feed Efficiency
MUFAs	Monounsaturated Fatty Acids
NEFAs	Non-Esterified Fatty Acids
OCFAs	Odd-Chain Fatty Acids
PCA	Principal Component Analysis
PUFAs	Polyunsaturated Fatty Acids
RFI	Residual Feed Intake
SFAs	Saturated Fatty Acids
sPLS-DA	Sparse Partial Least Squares Discriminant Analysis
TMR	Total Mixed Ration
VFAs	Volatile Fatty Acids

## References

[1] Chilliard Y., Ferlay A., Faulconnier Y., Bonnet M., Rouel J., Bocquier F. (2000). Adipose tissue metabolism and its role in adaptations to undernutrition in ruminants. Proc. Nutr. Soc..

[2] Jenkins T.C., Wallace R.J., Moate P.J., Mosley E.E. (2008). Board-invited review: Recent advances in biohydrogenation of unsaturated fatty acids within the rumen microbial ecosystem. J. Anim. Sci..

[3] Toral P.G., Hervás G., Fernández-Díez C., Belenguer A., Frutos P. (2021). Rumen biohydrogenation and milk fatty acid profile in dairy ewes divergent for feed efficiency. J. Dairy Sci..

[4] Zhang H., Elolimy A.A., Akbar H., Thanh L.P., Yang Z., Loor J.J. (2022). Association of residual feed intake with peripartal ruminal microbiome and milk fatty acid composition during early lactation in Holstein dairy cows. J. Dairy Sci..

[5] Carberry C.A., Kenny D.A., Han S., McCabe M.S., Waters S.M. (2012). Effect of phenotypic residual feed intake and dietary forage content on the rumen microbial community of beef cattle. Appl. Environ. Microbiol..

[6] Li B., Berglund B., Fikse W.F., Lassen J., Lidauer M.H., Mäntysaari P., Løvendahl P. (2017). Neglect of lactation stage leads to naive assessment of residual feed intake in dairy cattle. J. Dairy Sci..

[7] Atti N., Kayouli C., Mahouachi M., Guesmi A., Doreau M. (2002). Effect of a drastic and extended underfeeding on digestion in Barbary ewe. Anim. Feed Sci. Technol..

[8] Shingfield K.J., Bernard L., Leroux C., Chilliard Y. (2010). Role of *trans* fatty acids in the nutritional regulation of mammary lipogenesis in ruminants. Animal.

[9] Ahmad A.A., Yang C., Zhang J., Kalwar Q., Liang Z., Li C., Du M., Yan P., Long R., Han J. (2020). Effects of dietary energy levels on rumen fermentation, microbial diversity, and feed efficiency of yaks (*Bos grunniens*). Front. Microbiol..

[10] Gross J., Van Dorland H.A., Bruckmaier R.M., Schwarz F.J. (2011). Milk fatty acid profile related to energy balance in dairy cows. J. Dairy Res..

[11] Dórea J.R.R., French E.A., Armentano L.E. (2017). Use of milk fatty acids to estimate plasma nonesterified fatty acid concentrations as an indicator of animal energy balance. J. Dairy Sci..

[12] Khiaosa-ard R., Kleefisch M.T., Zebeli Q., Klevenhusen F. (2020). Milk fatty acid composition reflects metabolic adaptation of early lactation cows fed hay rich in water-soluble carbohydrates with or without concentrates. Anim. Feed Sci. Technol..

[13] Wilkinson J.M. (2011). Re-defining efficiency of feed use by livestock. Animal.

[14] Connor E.E., Hutchison J.L., Olson K.M., Norman H.D. (2012). Triennial lactation symposium: Opportunities for improving milk production efficiency in dairy cattle. J. Anim. Sci..

[15] Løvendahl P., Difford G.F., Li B., Chagunda M.G.G., Huhtanen P., Lidauer M.H., Lassen J., Lund P. (2018). Selecting for improved feed efficiency and reduced methane emissions in dairy cattle. Animal.

[16] Marinho M.N., Zimpel R., Peñagaricano F., Santos J.E.P. (2021). Assessing feed efficiency in early and mid lactation and its associations with performance and health in Holstein cows. J. Dairy Sci..

[17] Pires J.A.A., Larsen T., Leroux C. (2022). Milk metabolites and fatty acids as noninvasive biomarkers of metabolic status and energy balance in early-lactation cows. J. Dairy Sci..

[18] Marina H., Arranz J.J., Suárez-Vega A., Pelayo R., Gutiérrez-Gil B., Toral P.G., Hervás G., Frutos P., Fonseca P.A.S. (2024). Assessment of milk metabolites as biomarkers for predicting feed efficiency in dairy sheep. J. Dairy Sci..

[19] Barrio E., Hervás G., Gindri M., Friggens N.C., Toral P.G., Frutos P. (2023). Relationship between feed efficiency and resilience in dairy ewes subjected to acute underfeeding. J. Dairy Sci..

[20] Shingfield K.J., Ahvenjärvi S., Toivonen V., Äröla A., Nurmela K.V.V., Huhtanen P., Griinari J.M. (2003). Effect of dietary fish oil on biohydrogenation of fatty acids and milk fatty acid content in cows. Anim. Sci..

[21] de la Fuente M.A., Rodríguez-Pino V., Juárez M. (2015). Use of an extremely polar 100-m column in combination with a cyanoalkyl polysiloxane column to complement the study of milk fats with different fatty acid profiles. Int. Dairy J..

[22] Bichi E., Hervás G., Toral P.G., Loor J.J., Frutos P. (2013). Milk fat depression induced by dietary marine algae in dairy ewes: Persistency of milk fatty acid composition and animal performance responses. J. Dairy Sci..

[23] R Core Team (2024). R: A Language and Environment for Statistical Computing.

[24] Noguchi K., Gel Y.R., Brunner E., Konietschke F. (2012). nparLD: An R software package for the nonparametric analysis of longitudinal data in factorial experiments. J. Stat. Softw..

[25] Lenth R.V. (2016). Least-squares means: The R package lsmeans. J. Stat. Softw..

[26] Wickham H. (2011). ggplot2: Elegant Graphics for Data Analysis. WIREs Comput. Stat..

[27] Rohart F., Gautier B., Singh A., Le Cao K.A. (2017). mixOmics: An R package for ’omics feature selection and multiple data integration. PLoS Comput. Biol..

[28] Barrio E., Frutos P., Friggens N.C., Toral P.G., Hervás G. (2025). Feed efficiency and resilience in dairy ewes subjected to a nutritional challenge. J. Dairy Sci..

[29] Vlaeminck B., Fievez V., Cabrita A.R.J., Fonseca A.J.M., Dewhurst R.J. (2006). Factors affecting odd- and branched-chain fatty acids in milk: A review. Anim. Feed Sci. Technol..

[30] Artegoitia V.M., Foote A.P., Lewis R.M., Freetly H.C. (2017). Rumen fluid metabolomics analysis associated with feed efficiency on crossbred steers. Sci. Rep..

[31] Rukkwamsuk T., Geelen M.J.H., Druip T.A.M., Wensing T. (2000). Interrelation of fatty acid composition in adipose tissue, serum, and liver of dairy cows during the development of fatty liver postpartum. J. Dairy Sci..

[32] Bernard L., Leroux C., Chilliard Y. (2013). Expression and nutritional regulation of stearoyl-CoA desaturase genes in the ruminant mammary gland: Relationship with milk fatty acid composition. Stearoyl-CoA Desaturase Genes in Lipid Metabolism.

[33] Lisuzzo A., Fiore F., Harvatine K., Mazzotta E., Berlanda M., Spissu N., Badon T., Contiero B., Moscati L., Fiore E. (2022). Changes in plasma fatty acids profile in hyperketonemic ewes during early lactation: A preliminary study. Sci. Rep..

[34] Tsiplakou E., Chadio S., Papadomichelakis G., Zervas G. (2012). The effect of long term under- and over-feeding on milk and plasma fatty acids profile and on insulin and leptin concentrations of goats. Int. Dairy J..

[35] Orquera-Arguero K.G., Villalba D., Blanco M., Ferrer J., Casasús I. (2022). Modelling beef cows’ individual response to short nutrient restriction in different lactation stages. Animal.

[36] Atti N., Bocquier F., Khaldi G. (2004). Performance of the fat-tailed Barbarine sheep in its environment: Adaptive capacity to alternation of underfeeding and re-feeding periods. Anim. Res..

[37] Chilliard Y., Toral P.G., Shingfield K.J., Rouel J., Leroux C., Bernard L. (2014). Effects of diet and physiological factors on milk fat synthesis, milk fat composition and lipolysis in the goat: A short review. Small Rumin. Res..

[38] Toral P.G., Hervás G., Frutos P. (2018). Use of high doses of 18:0 to try to mitigate the syndrome of milk fat depression in dairy ewes fed marine lipids. Anim. Feed Sci. Technol..

[39] Dewanckele L., Toral P.G., Vlaeminck B., Fievez V. (2020). Role of rumen biohydrogenation intermediates and rumen microbes in diet-induced milk fat depression: An update. J. Dairy Sci..

[40] Bell A.W. (1995). Regulation of organic nutrient metabolism during transition from late pregnancy to early lactation. J. Anim. Sci..

[41] Bauman D.E., Griinari J.M. (2003). Nutritional regulation of milk fat synthesis. Annu. Rev. Nutr..

[42] Fulco A.J. (1983). Fatty acid metabolism in bacteria. Prog. Lipid Res..

[43] Kaneda T. (1991). *Iso*- and *anteiso*-fatty acids in bacteria: Biosynthesis, function, and taxonomic significance. Microbiol. Rev..

[44] Vlaeminck B., Gervais R., Rahman M.M., Gadeyne F., Gorniak M., Doreau M., Fievez V. (2015). Postruminal synthesis modifies the odd- and branched-chain fatty acid profile from the duodenum to milk. J. Dairy Sci..

[45] Hocquette J.F., Bauchart D. (1999). Intestinal absorption, blood transport and hepatic and muscle metabolism of fatty acids in preruminant and ruminant animals. Reprod. Nutr. Dev..

[46] Drackley J.K., Overton T.R., Douglas G.N. (2001). Adaptations of glucose and long-chain fatty acid metabolism in liver of dairy cows during the periparturient period. J. Dairy Sci..

[47] Jakobsson A., Westerberg R., Jacobsson A. (2006). Fatty acid elongases in mammals: Their regulation and roles in metabolism. Prog. Lipid Res..

[48] Drouin G., Rioux V., Legrand P. (2019). The n-3 docosapentaenoic acid (DPA): A new player in the n-3 long chain polyunsaturated fatty acid family. Biochimie.

[49] Moallem U. (2018). Roles of dietary n-3 fatty acids in performance, milk fat composition, and reproductive and immune systems in dairy cattle. J. Dairy Sci..

